# Endothelial adenosine A_2A_ receptor activation-mediated Tau hyperphosphorylation leads to blood–brain barrier breakdown in a chronic methamphetamine mouse model

**DOI:** 10.3389/fmed.2026.1801011

**Published:** 2026-04-07

**Authors:** Ying Qian, Meigui Hu, Xingxing Xie, Li Ren, Xiaorong Huang, Shanshan Hu

**Affiliations:** 1 Department of Pharmacy, Affiliated Hospital of Zunyi Medical University, Zunyi, China; 2 Department of Pulmonary and Critical Care Medicine, The Fourth People's Hospital of Guiyang City, Guiyang, China; 3 West China School of Pharmacy, Sichuan University, Chengdu, China; 4 Department of Pharmacy, Ya'an People's Hospital, Ya'an, China; 5 Good Clinical Practice Center, Affiliated Hospital of Zunyi Medical University, Zunyi, China

**Keywords:** adenosine receptor, blood brain barrier, methamphetamine, microtubule associated protein tau, microvessel endothelial cell

## Abstract

**Introduction:**

Methamphetamine (METH), a psychostimulant, can cause the blood–brain barrier (BBB) breakdown through astrocyte endfeet swelling and endothelial cell impairment. Our previous studies have shown that phosphorylated microtubule-associated protein Tau (p-Tau) levels increase, especially in neurons, after METH exposure. However, whether p-Tau accumulates in the endothelial cells of METH mouse brains remains unclear. The effects of endothelial p-Tau accumulation on BBB integrity in a chronic METH mouse model are elusive.

**Methods:**

The A_2A_R-GSK3β signaling pathway protein levels were evaluated in chronic METH mice model. The BBB integrity, endothelial p-Tau level and behavioral performances were tested after A_2A_R inhibiton, GSK3β inhibition or p-Tau knockout.

**Results:**

In this study, we found that METH might induce the upregulation of adenosine receptor subtype 2A (A_2A_R), leading to glycogen synthase kinase-3 beta (GSK3β) activation and Tau phosphorylation in BBB endothelial cells. Chronic METH exposure induced BBB breakdown and anxiety- and depression-like behavioral abnormalities. Pharmacological inhibition of A_2A_R and GSK3β activation alleviated p-Tau accumulation, mitigated the behavioral changes, and alleviated BBB destruction induced by METH. Moreover, genetically knocking out Tau also attenuated BBB destruction and behavioral changes induced by chronic METH.

**Discussion:**

Based on these findings, we propose an A_2A_R-GSK3β signaling-dependent mechanism to elucidate METH-induced BBB breakdown. We suggest p-Tau as a promising candidate target to reduce BBB destruction and behavioral abnormalities in METH abusers.

## 1 Introduction

Methamphetamine (METH), a psychostimulant, possesses a chemical structure similar to dopamine; thus, it selectively injures the dopaminergic neurons (1). Recent studies have shown that METH might damage other types of neurons, such as serotonergic neurons and GABAergic interneurons (2). METH might induce neurodegeneration through neuroinflammation, mitochondrial autophagy deficit, and endoplasmic reticulum stress (3). Recent studies have highlighted the impairment of the blood–brain barrier (BBB) as a critical event in METH-induced neurodegeneration, whereas manipulations of the BBB may be beneficial (4, 5).

The BBB is composed of astrocyte endfeet, endothelial tight junctions, basement membranes, and pericytes (6). It selectively regulates the transport of substances between the plasma and the brain parenchyma (7, 8). Brain microvascular endothelial cells, which are located on the luminal side of the BBB, express an abundance of transporters for cytokines and metabolic hormones (9). Moreover, membranous receptors are also expressed on the endothelial cells to sense the environment of the blood (10). Among these receptors, the adenosine receptor (AR) has gained much attention in recent years (11). The AR has four subtypes: A_1_R, A_2A_R, A_2B_R, and A_3_R. The brain A_2A_Rs are largely activated in brain injury and neurodegenerative diseases (12). A_2A_R, a type of G protein-coupled receptor, can activate the Cyclic adenosine monophosphate/protein kinase A (cAMP/PKA) and glycogen synthase kinase-3 beta (GSK3β) pathways (13). Endothelial A_2A_R activation induces cognitive impairment in an insulin-resistant mouse model (14). Previous studies have also found that A_2A_R blockade ameliorated cerebrovascular dysfunction. A recent study has demonstrated that A_2A_R inactivation by caffeine or the knockout of A_2A_R alleviated cognitive dysfunction in a traumatic brain injury mouse model (15). Our previous study has shown that METH can disrupt BBB integrity through α-synuclein (4). However, whether A_2A_R is involved in METH-induced BBB breakdown remains elusive.

The microtubule-associated protein Tau (MAP-Tau, Tau) has been implicated in Alzheimer’s disease (AD) and is a target for treating AD progression (16). In our previous studies, we found that Tau was hyperphosphorylated in the prefrontal cortex, the hippocampus, the striatum, and the midbrain in the METH mouse model (17). At present, there are no studies elaborating on the relationship between endothelial A_2A_R and phosphorylated Tau (p-Tau) in the METH mouse model. We hypothesize that endothelial p-Tau accumulation might trigger endothelial pathology and dysfunction, leading to BBB disruption.

Previous studies have shown that reduced BBB integrity is observed in a social stress-induced depression mouse model, and downregulation of claudin-5, a tight junction (TJ) protein, was sufficient to induce depression-like behaviors (18). METH can induce many psychiatric disorders, including depression, anxiety, and addiction (19, 20). Based on the above studies, we propose that METH-induced BBB disruption could trigger depression- and anxiety-like behaviors.

In our study, we established a chronic METH model to study the role of the A_2A_R-GSK3β pathway in BBB integrity. We found that the BBB was disrupted, and endothelial tight junction (TJ) proteins were reduced in this model. Moreover, we discovered that the A_2A_R-GSK3β signaling was activated, and BBB endothelial cellular Tau was hyperphosphorylated, leading to behavioral abnormalities. Pharmacological inhibition of A_2A_R or GSK3β reduced the p-Tau levels in brain microvascular endothelial cells and ameliorated BBB breakdown induced by METH. Genetically knocking out Tau also alleviated BBB disruption and behavioral changes induced by METH.

## 2 Materials and methods

### 2.1 Animals and experimental scheme

Wild-type (WT) C57BL/6J male mice (weighing 20–25 g, aged approximately 4 weeks old) were purchased from the laboratory animal center of Zunyi Medical University. The Tau knockout mice (B6.129 × 1-*Mapt*^tm/Hnd^/J, *mapt*^−/−^, approximately 4 weeks old) were purchased from the Jackson Laboratory (Bar Harbor, ME, USA). Those mice were housed in a controlled environment (temperature: 22 ± 2 °C, humidity: 50–55%). Animal procedures were performed according to the National Institutes of Health (NIH) Guide for Care and Use and were approved in advance by the Zunyi Medical University Institutional Animal Care and Use Committee. The METH chronic exposure model was selected to mimic the pattern of long-term METH use in humans.

In the first phase, the WT mice were randomly divided into four groups: Con: saline was administered intraperitoneally in place of METH; Con+SCH: the A_2A_R antagonist SCH58261 (0.1 mg/kg in saline) was administered intraperitoneally for 3 days, once daily from day 12 to day 14; METH: METH was administered intraperitoneally from day 1 to day 14 (Table 1); and METH+SCH: METH was administered as shown in Table 1, and the A_2A_R antagonist SCH58261 was administered (0.1 mg/kg in saline) intraperitoneally for 3 days, once daily from day 12 to day 14.

**Table 1:** Dosing schedule of methamphetamine (METH) treatment (mg/kg).

Day	1	2	3	4	5	6	7	8	9	10	11	12	13	14
8:00	1.0	1.0	1.0	1.0	1.5	1.5	2.0	2.0	2.5	3.0	3.5	4.0	4.5	5.0
10:00				1.0	1.5	1.5	2.0	2.0	2.5	3.0	3.5	4.0	4.5	5.0
12:00				1.0	1.5	1.5	2.0	2.0	2.5	3.0	3.5	4.0	4.5	5.0
14:00		1.0	1.0	1.0	1.5	1.5	2.0	2.0	2.5	3.0	3.5	4.0	4.5	5.0

In the second phase, the WT mice were randomly divided into four groups: Con: saline was administered intraperitoneally in place of METH; Con+LiCl: the GSK3β inhibitor lithium chloride (LiCl, 40 mg/kg in saline) was administered intraperitoneally for 3 days, once daily from day 12 to day 14; METH: METH was administered intraperitoneally from day 1 to day 14 (Table 1); and METH+LiCl: METH was administered as shown in Table 1, and the GSK3β inhibitor lithium chloride (LiCl, 40 mg/kg in saline) was administered intraperitoneally for 3 days, once daily from day 12 to day 14.

In the third phase, the WT mice and Tau knockout mice were used and divided into four groups: Con: saline was administered intraperitoneally to WT mice in place of METH; Tau KO: saline was administered intraperitoneally to Tau KO mice in place of METH; WT + METH: METH was administered intraperitoneally to WT mice, as shown in Table 1; and Tau KO + METH: METH was administered intraperitoneally to Tau KO mice, as shown in Table 1.

### 2.2 Immunofluorescence (IF) staining

Mouse tissues were acquired before deep anesthesia (with ketamine: 120 mg/kg and xylazine: 8 mg/kg, intraperitoneally). Mouse brains were acquired and fixed in 4% Paraformaldehyde (PFA) for 24 h. Then, the mouse brains were sectioned (40 μm in thickness) using a microtome (CM1950, Leica, Wetzlar, Germany). Brain sections were incubated with 5% Bovine Serum Albumin (BSA) and 0.5% Triton X-100 in phosphate-buffered saline (PBS) for 2 h. The sections were incubated with the primary antibodies A_2A_R (1:200 dilution, Cat# ab3461, Abcam, MA, USA), phosphor S396 Tau (1:200 dilution, Cat# ab109390, Abcam, MA, USA), and CD31 (1:200 dilution, Cat# 3528, CST, Danvers, MA, USA) overnight at 4 °C. Adequate secondary antibodies were incubated for 1 h. Nuclei were labeled with 4, 6-diamidino-2-phenylindole (DAPI) (Cat# H-1020, Vector Lab, Burlingame, CA, USA). Images were taken using a laser scanning confocal microscope (FV3000, Nikon, Japan). Images were analyzed using ImageJ (NIH).

### 2.3 Transmission electron microscope (TEM) analysis

Mouse hippocampal tissues were detached and fixed in 2.5% glutaraldehyde for 4 h. After infiltrating the tissues in Epon resin overnight, the ultrathin sections were prepared using an ultramicrotome. The sections were picked up on grids and stained with lead citrate. The images were taken using an electron microscope (Tecnai G2, Thermo Fisher Scientific, MA, USA) equipped with a charge-coupled device (CCD) camera.

### 2.4 Immunoblotting

Hippocampal tissues were homogenized to collect the protein supernatant, which was then mixed with a loading buffer to obtain protein samples. The samples were separated by electrophoresis and transferred to polyvinylidene fluoride (PVDF) membranes, which were then blocked and incubated with primary antibodies: A2AR (1:1,000 dilution, Cat#A1587, ABclonal, China), p-GSK3β Y216 (1:1,000 dilution, Cat#ab68476, Abcam, USA), p-Ser396 Tau (1:1,000 dilution, Cat#9632S, CST), Tau (1:1,000 dilution, Cat#ab80579, Abcam, MA, USA), claudin-5 (Cat#35-2500, 1:1,000, Invitrogen), occludin (1:1,000 dilution, Cat#ab216327, Abcam, MA, USA), and β-actin (1:1,000 dilution, Cat#ab8226, Abcam, MA, USA). Following incubation with secondary antibodies, the membranes’ signals were visualized using enhanced chemiluminescence Electrochemiluminescence (ECL) reagents (Cat#170-5061, Bio-Rad, USA). Protein expression levels were normalized to the β-actin intensity.

### 2.5 Behavioral testing

For the open field test (OFT), a video camera was placed above a chamber (40 × 40 × 30 cm). Mice were placed in the chamber to explore the area for 5 min. The video was analyzed using the software (VisuTrack 3.0, Xin Luan MDT Infotech Ltd., Shanghai, China).

For the forced swimming test (FST), a cylinder-shaped glass container (diameter: 19 cm, height: 40 cm) was filled with water (23–25 °C). Mice were placed in the container for 5 min. The duration of immobility was recorded by an investigator who was blinded to the experiment.

### 2.6 Statistical analysis

Data were expressed as mean ± standard error of mean (M ± SEM). For comparisons between two groups, the two-tailed unpaired Student’s *t*-test was used. A one-way analysis of variance (ANOVA) followed by Bonferroni’s multiple comparison test was used to compare multiple groups using SPSS version 22.0 (IBM, NY, USA). Data were plotted using GraphPad 8 (GraphPad, USA). Significance was accepted at a *p-*value of < 0.05. Statistical parameters, including *T*, *F*, and *p* values, and analytical methods used for each analysis are listed in the figure legends.

## 3 Results

### 3.1 METH-induced A_2A_R-GSK3β activation and endothelial p-Tau accumulation

**Figure 1:**
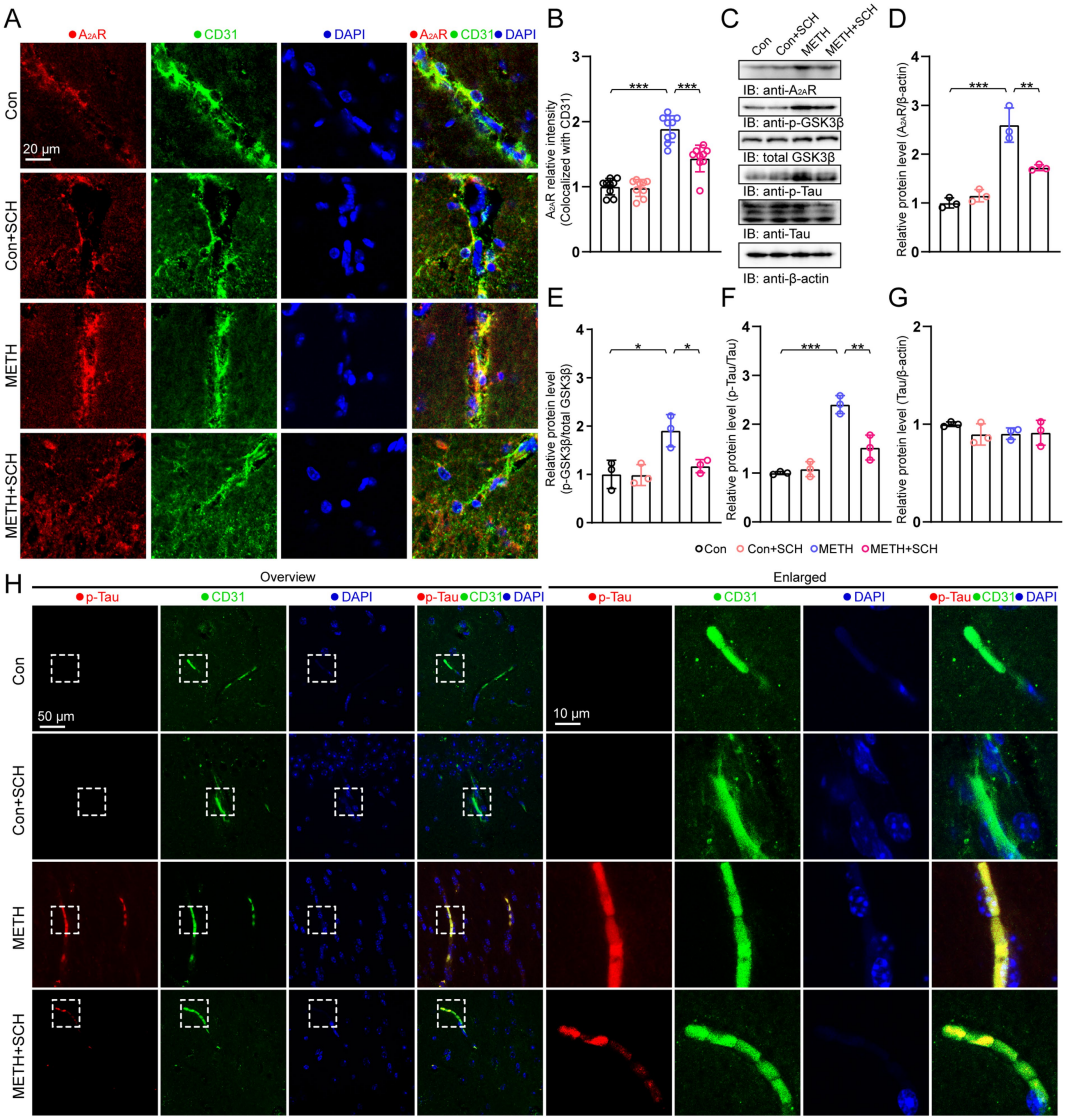
Endothelial A_2A_R activation mediated Tau hyperphosphorylation in the chronic METH model. **(A)** IF staining of A_2A_R and CD31 in brain sections. **(B)** Relative intensity of A_2A_R colocalization with CD31. Data were analyzed using a one-way ANOVA followed by Bonferroni’s *post-hoc* test. *F*(3, 26) = 57.156, *p* < 0.001, *n* = 9. **(C)** Immunoblots of A_2A_R, p-GSK3β, p-Tau, Tau, and β-actin. **(D)** Quantification of A_2A_R normalized to β-actin. Data were analyzed using a one-way ANOVA followed by Bonferroni’s *post-hoc* test. *F*(3, 11) = 40.704, *p* < 0.001, *n* = 3. **(E)** Quantification of p-GSK3β normalized to total GSK3β. Data were analyzed using a one-way ANOVA followed by Bonferroni’s *post-hoc* test. *F*(3, 11) = 8.621, *p* = 0.007, *n* = 3. **(F)** Quantification of p-Tau normalized to total Tau. Data were analyzed using a one-way ANOVA followed by Bonferroni’s *post-hoc* test. *F*(3, 11) = 40.228, *p* < 0.001, *n* = 3. **(G)** Quantification of Tau normalized to β-actin. Data were analyzed using a one-way ANOVA followed by Bonferroni’s *post-hoc* test. *F*(3, 11) = 0.87, *p* = 0.496, *n* = 3. **(H)** IF staining of p-Tau and CD31. *p < 0.05, **p < 0.01, ***p < 0.001.

We first detected the A_2A_R in endothelial cells using immunostaining. We found that the intensity of A_2A_R (colocalized with CD31) was increased in the METH group compared with that in the control group. In this study, we used SCH58261 (SCH) to specifically inhibit the A_2A_R. We revealed that the A_2A_R expression was lower in the METH+SCH group than in the METH group (Figures 1A,B). Immunoblotting results showed that METH-induced A_2A_R, p-GSK3β, and p-Tau levels were increased. Intervention with SCH significantly reduced the levels of A_2A_R, p-GSK3β, and p-Tau in the METH group mice (Figures 1C–G). We also performed an immunofluorescence (IF) of CD31 and p-Tau and found a similar result to the immunoblots. The colocalized intensity of CD31 and p-Tau was higher in the METH group than in the control group, and SCH treatment diminished the increase (Figure 1H).

### 3.2 A_2A_R inhibition alleviated METH-triggered BBB breakdown and behavioral abnormalities

**Figure 2:**
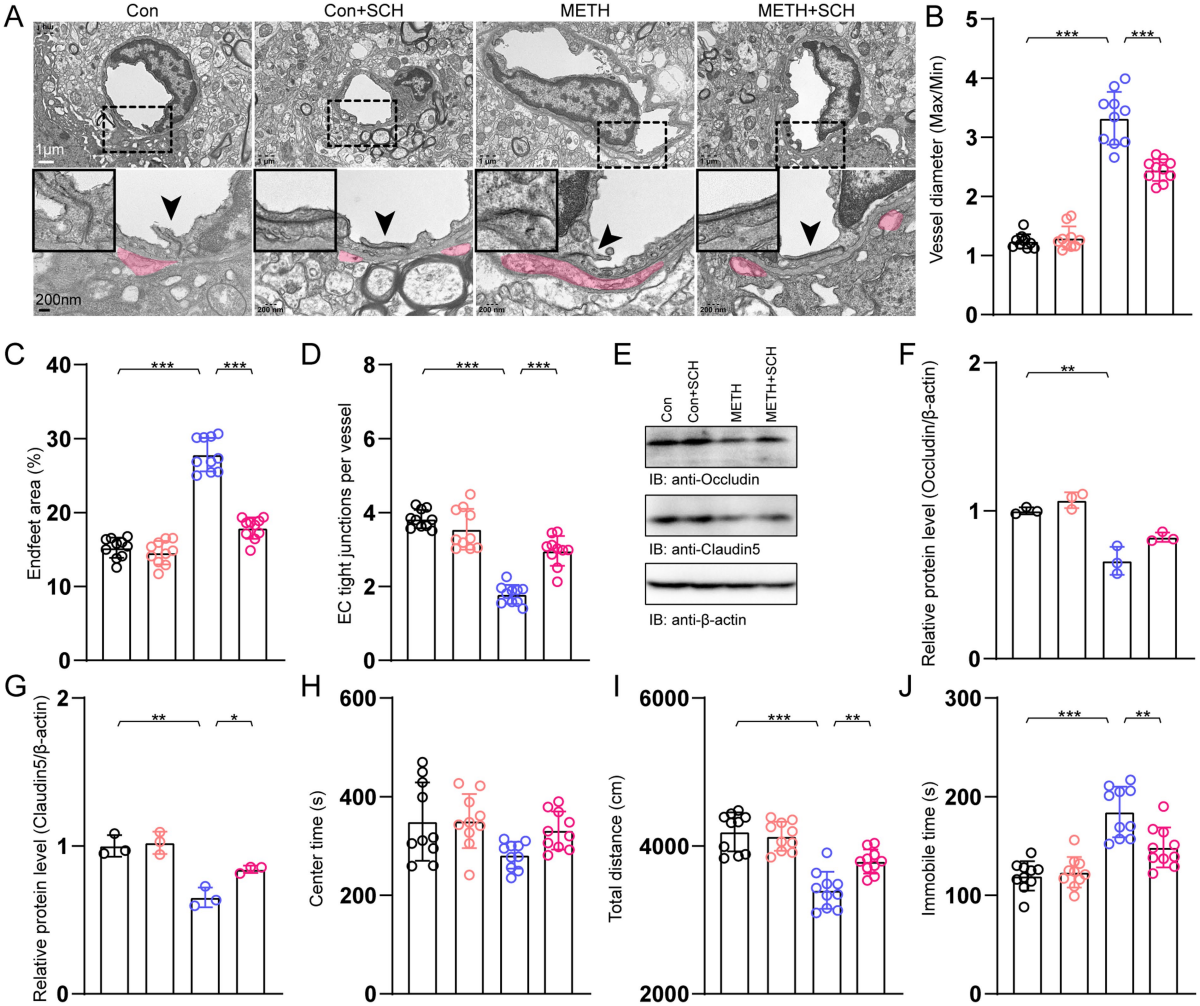
Effects of A_2A_R on BBB integrity, TJ protein levels, and behavioral performance. **(A)** TEM analysis of the BBB. Arrowheads indicated TJs in the lower panel. The magnified pictures in the corners indicated the TJs of the arrowhead areas. Red-shaded areas indicated the astrocyte endfeet areas. **(B)** The analysis of vessel diameter (max/min). Data were analyzed using a one-way ANOVA followed by Bonferroni’s *post-hoc* test. *F*(3, 29) = 140.4, *p* < 0.01, *n* = 10. **(C)** Quantification of astrocyte end-feet area. Data were analyzed using a One-way ANOVA followed by Bonferroni’s *post-hoc* test. *F*(3, 29) = 129.036, *p* < 0.01, *n* = 10. **(D)** Quantification of the TJ number per vessel. Data were analyzed using a one-way ANOVA followed by Bonferroni’s *post-hoc* test. *F*(3, 29) = 55.11, *p* < 0.01, *n* = 10. **(E)** Immunoblots of TJ proteins occludin and claudin-5. **(F)** Quantification of occludin normalized to β-actin. Data were analyzed using a one-way ANOVA followed by Bonferroni’s *post-hoc* test. *F*(3, 11) = 30.19, *p* < 0.01, *n* = 3. **(G)** Quantification of claudin-5 normalized to β-actin. Data were analyzed using a one-way ANOVA followed by Bonferroni’s *post-hoc* test. *F*(3, 11) = 21.77, *p* < 0.01, *n* = 3. **(H)** Analysis of center time in the OFT. Data were analyzed using a one-way ANOVA followed by Bonferroni’s *post-hoc* test. *F*(3, 11) = 1.42, *p* = 0.253, *n* = 3. **(I)** Analysis of total distance in the OFT. Data were analyzed using a one-way ANOVA followed by Bonferroni’s *post-hoc* test. *F*(3, 11) = 26.815, *p* < 0.01, *n* = 10. **(J)** Analysis of immobile time in the FST. Data were analyzed using a one-way ANOVA followed by Bonferroni’s *post-hoc* test. *F*(3, 11) = 23.35, *p* < 0.01, *n* = 10. *p < 0.05, **p < 0.01, ***p < 0.001.

To determine whether METH might trigger BBB breakdown, we conducted a TEM analysis. The results showed a larger vessel diameter ratio (max/min), a higher endfeet area percentage, and a lower number of TJ proteins after METH exposure. Intervention with SCH reduced these changes (Figures 2A–D). Next, we tested the TJ proteins, including occludin and claudin-5. Immunoblotting showed that the occludin and claudin-5 were decreased (approximately 33.8 and 34.8%, respectively) in the METH group compared with that in the control group (Figures 2E–G). Compared with the METH group, the occludin and claudin-5 levels were increased after SCH treatment in the METH+SCH group.

Next, we tested METH-induced behavioral changes using the OFT and the FST. We found that the center time and the total distance were decreased (approximately 13.6 and 18.9%, respectively) in the METH group compared with that in the control group (Figures 2H,I). SCH treatment diminished these decreases. The increased immobile time induced by METH was also attenuated by SCH treatment (Figure 2J).

### 3.3 Inhibiting GSK3β activation ameliorated endothelial tau phosphorylation

**Figure 3:**
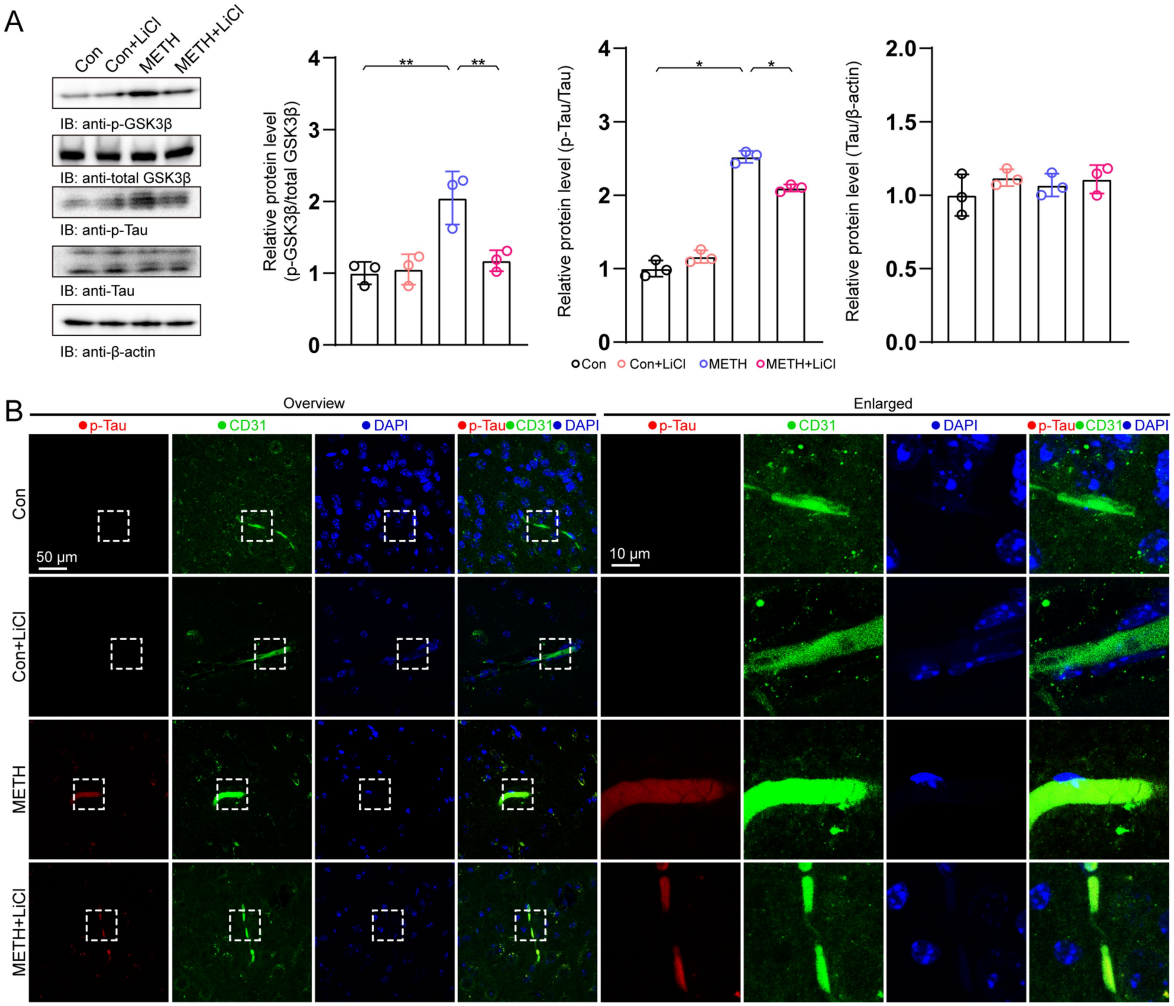
GSK3β inhibition reduced endothelial p-Tau levels in the METH mouse model. **(A)** Immunoblots and quantification of p-GSK3β, p-Tau, Tau, and β-actin. p-GSK3β/GSK3β, one-way ANOVA with Bonferroni’s *post-hoc* test. *F*(3, 11) = 12.71, *p* = 0.02, *n* = 3. p-Tau/Tau, one-way ANOVA with Bonferroni’s *post-hoc* test. *F*(3, 11) = 225.63, *p* < 0.001, *n = 3. Tau/β-actin*, one-way ANOVA with Bonferroni’s *post-hoc* test. *F*(3, 11) = 0.901, *p* = 0.901, *n* = 3. **(B)** IF staining of A_2A_R and CD31 in hippocampal areas. *p < 0.05, **p < 0.01, ***p < 0.001.

Next, we investigated the role of GSK3β in Tau phosphorylation level using lithium chloride (LiCl, a specific GSK3β inhibitor). Immunoblotting results showed that LiCl treatment reduced METH-induced p-GSK3β increasing by approximately 36.7% (Figure 3A). Moreover, the p-Tau level was also decreased (approximately 16.8%) in the METH+LiCl group compared with that in the METH group (Figure 3A). Notably, the total Tau was not comparable among the four groups. The immunostaining results also showed that the p-Tau, which colocalized with CD31, was also decreased in the METH+LiCl group compared with that in the METH group (Figure 3B).

### 3.4 Pharmacological inhibition of GSK3β alleviated BBB disruption and behavioral abnormalities in METH mice

**Figure 4:**
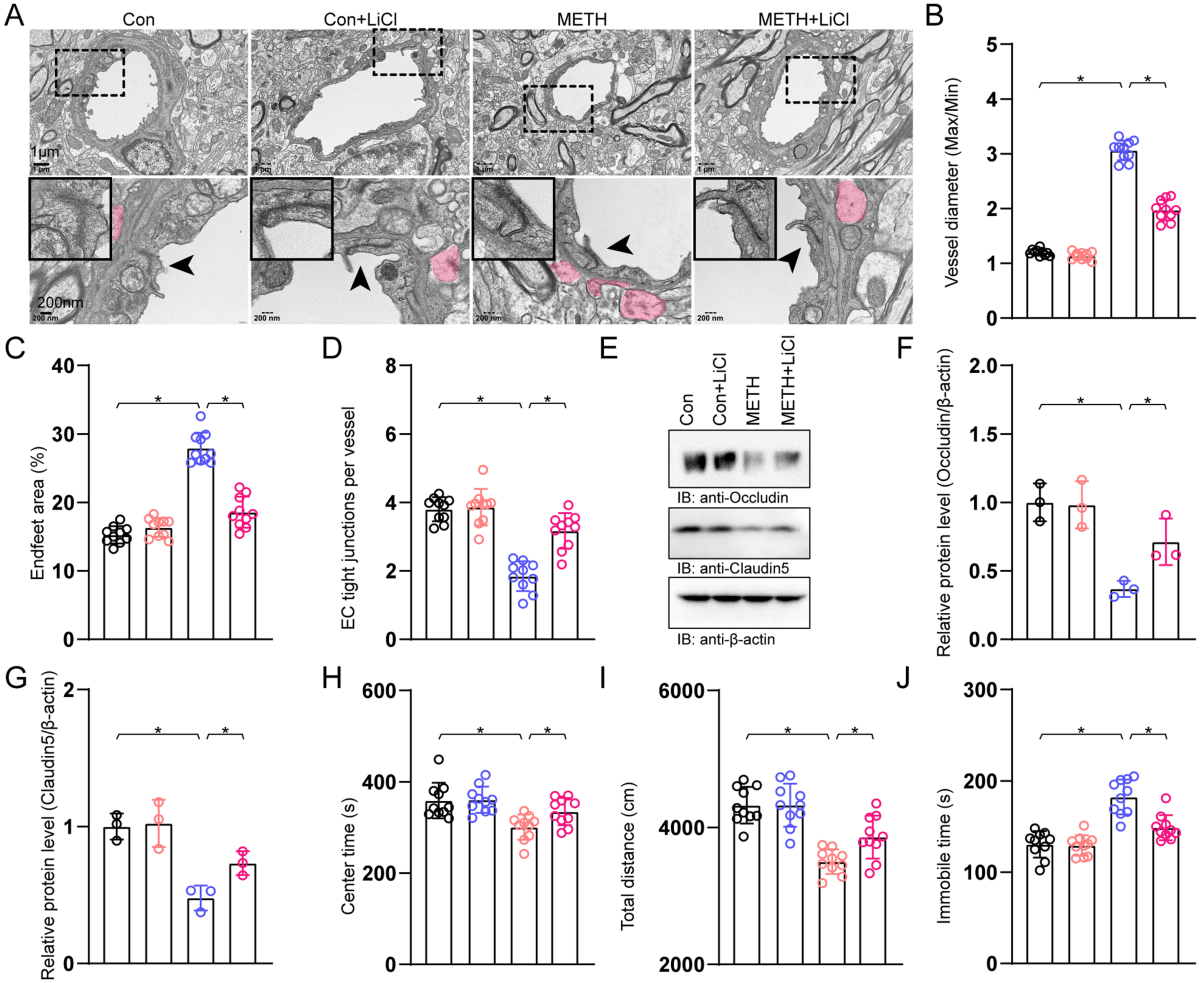
GSK3β inhibition ameliorated BBB disruption. **(A)** TEM analysis of the BBB. Arrowheads indicated TJs in the lower panel. The magnified pictures in the corners show the TJs of the arrowhead areas. Red-shaded areas indicated the astrocyte end-feet areas. **(B)** Analysis of vessel diameter (max/min). One-way ANOVA with Bonferroni’s *post-hoc* test. *F*(3, 29) = 424.858, *p* < 0.01, *n* = 10. **(C)** Quantification of astrocyte end-feet area. A one-way ANOVA with Bonferroni’s *post-hoc* test. *F*(3, 29) = 96.71, *p* < 0.01, *n* = 10. **(D)** Quantification of the TJ number per vessel. One-way ANOVA with Bonferroni’s *post-hoc* test. *F*(3, 29) = 40.61, *p* < 0.01, *n* = 10. **(E)** Immunoblots of TJ proteins occludin and claudin-5. **(F)** Quantification of occludin normalized to β-actin. A one-way ANOVA with Bonferroni’s *post-hoc* test. *F*(3, 11) = 12.96, *p* = 0.002, *n* = 3. **(G)** Quantification of claudin-5 normalized to β-actin. One-way ANOVA with Bonferroni’s *post-hoc* test. *F*(3, 11) = 14.486, *p* = 0.001, *n* = 3. **(H)** Analysis of center time in the OFT. A one-way ANOVA with Bonferroni’s *post-hoc* test. *F*(3, 11) = 7.863, *p* < 0.01, *n* = 3. **(I)** Analysis of the total distance in the OFT. A one-way ANOVA with Bonferroni’s *post-hoc* test. *F*(3, 11) = 21.265, *p* < 0.01, *n* = 10. **(J)** Analysis of immobile time in the FST. One-way ANOVA with Bonferroni’s *post-hoc* test. *F*(3, 11) = 27.337, *p* < 0.01, *n* = 10. *p < 0.05, **p < 0.01, ***p < 0.001.

We used a TEM analysis to observe the effects of GSK3β inhibition on BBB integrity. We found a larger vessel diameter (max/min), a higher endfeet coverage, and a lower TJ protein in mice treated with METH. An intervention with a GSK3β inhibitor diminished these changes in METH mice (Figures 4A–D). Moreover, the decreases in TJ proteins, including occludin and claudin-5, were attenuated by LiCl (Figures 4E–G). In the OFT, reductions in the center time and the total distance were also ameliorated by LiCl in METH mice (Figures 4H,I). In the FST, an increase in immobile time was diminished by LiCl in METH mice (Figure 4J).

### 3.5 Knocking out Tau diminished BBB pathology and behavioral changes induced by METH

**Figure 5:**
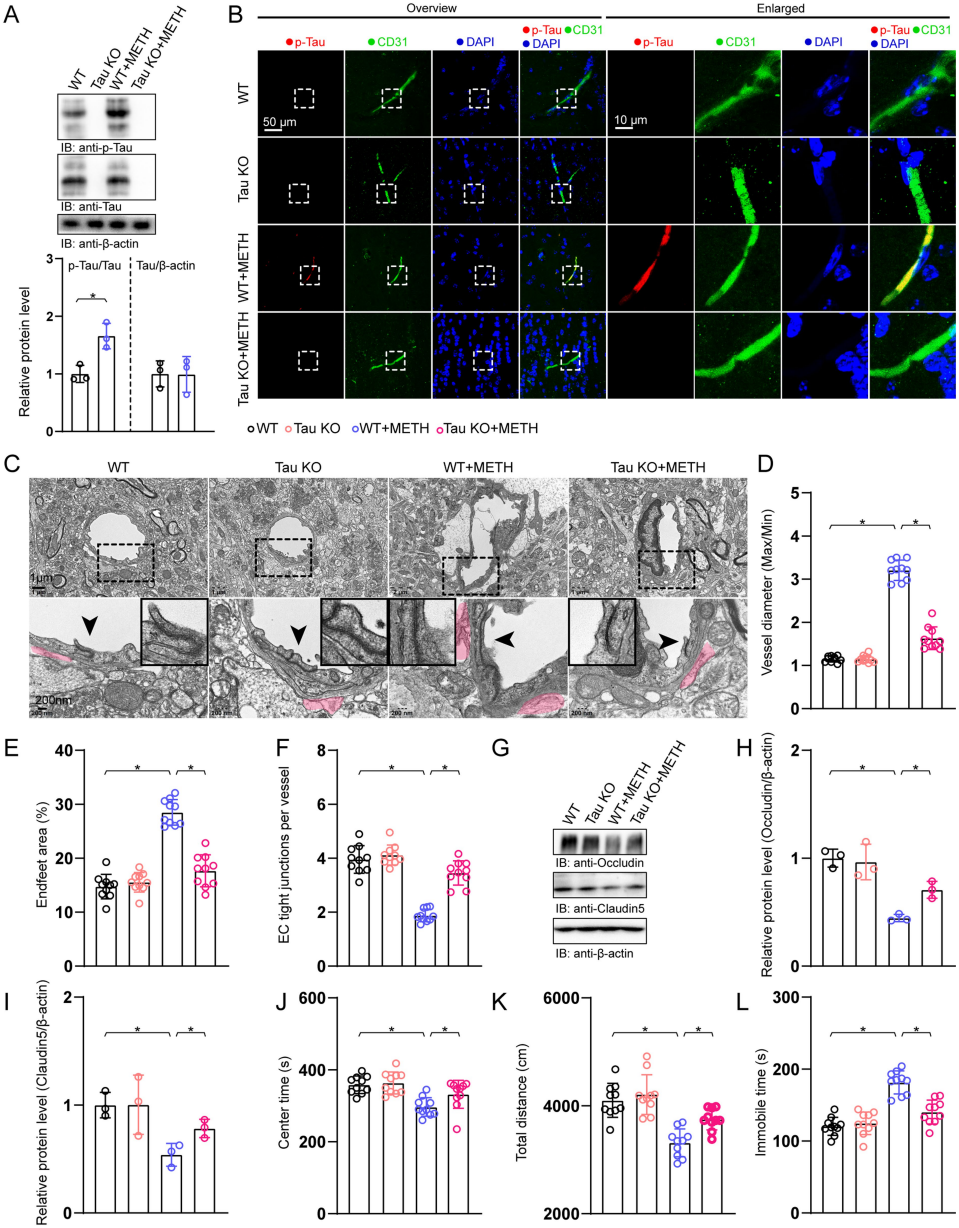
Knockout of Tau alleviated BBB breakdown and anxiety- and depression-like behaviors in METH mice. **(A)** Immunoblots and quantification of p-Tau, Tau, and β-actin. p-Tau, *T*-test, *p* = 0.515, Tau, *T*-test, *p* = 0.48, *n* = 3. **(B)** IF staining of A_2A_R and CD31 in hippocampal areas. **(C)** TEM analysis of the BBB. Arrowheads indicated TJs in the lower panel. The magnified pictures in the corners indicated the TJs of the arrowhead areas. Red-shaded areas indicated the astrocyte end-feet areas. **(D)** Analysis of vessel diameter (max/min). Data were analyzed using a one-way ANOVA followed by Bonferroni’s *post-hoc* test. *F*(3, 29) = 282.358, *p* < 0.01, *n* = 10. **(E)** Quantification of astrocyte end-feet area. A one-way ANOVA with Bonferroni’s *post-hoc* test. *F*(3, 29) = 71.629, *p* < 0.01, *n* = 10. **(F)** Quantification of the TJ number per vessel. A one-way ANOVA with Bonferroni’s *post-hoc* test. *F*(3, 29) = 64.285, *p* < 0.01, *n* = 10. **(G)** Immunoblots of TJ proteins occludin and claudin-5. **(H)** Quantification of occludin normalized to β-actin. A one-way ANOVA with Bonferroni’s *post-hoc* test. *F*(3, 11) = 19.304, *p* < 0.01, *n* = 3. **(I)** Quantification of claudin-5 normalized to β-actin. A one-way ANOVA with Bonferroni’s *post-hoc* test. *F*(3, 11) = 5.416, *p* < 0.01, *n* = 3. **(J)** Analysis of center time in the OFT. A one-way ANOVA with Bonferroni’s *post-hoc* test. *F*(3, 29) = 10.069, *p* < 0.01, *n* = 3. **(K)** Analysis of the total distance in the OFT. A one-way ANOVA with Bonferroni’s *post-hoc* test. *F*(3, 29) = 19.136, *p* < 0.01, *n* = 10. **(L)** Analysis of immobile time in the FST. A one-way ANOVA with Bonferroni’s *post-hoc* test. *F*(3, 29) = 33.136, *p* < 0.01, *n* = 10. *p < 0.05, **p < 0.01, ***p < 0.001.

Immunoblotting and immunostaining results showed that the p-Tau was not tested in the Tau KO group and the Tau KO + METH group (Figures 5A,B). We next investigated the effects of knocking out Tau on BBB integrity and behavioral performances. An ultrastructural analysis of the BBB showed that increased vessel diameter (max/min), the increased endfeet coverage, and reduced number of TJ proteins were ameliorated by genetic knockout of Tau in METH mice (Figures 5C–F). The reductions in TJ protein levels induced by METH were attenuated by genetic knockout of Tau (Figures 5G–I). The decreases in the center time and the total distance were also ameliorated by Tau knockout in METH mice (Figures 5J,K). The FST results showed that an increase in immobile time induced by METH was diminished by genetic knockout of Tau (Figure 5L).

## 4 Discussion

METH might induce BBB breakdown through multiple pathways, including astrocyte activation and endothelial cell impairment (21). The endothelial cell and TJs were vulnerable to METH (22). Thus, in this study, we focused on the effects of METH on endothelial cells and the effects of A_2A_R-GSK3β signaling on endothelial cellular function. We found that METH induced endothelial cell p-Tau accumulation through A_2A_R-GSK3β signaling, leading to BBB breakdown and TJ protein loss. The depression- and anxiety-like behaviors were observed in this chronic METH model. Pharmacological blocking of A_2A_R-GSK3β or genetic knockout of Tau alleviated BBB destruction and behavioral abnormalities induced by METH.

The cellular A_2A_R-GSK3β signaling has been shown to be involved in the pathophysiological mechanism of neurodegenerative diseases, such as Parkinson’s disease (PD), Alzheimer’s disease (AD), and experimental autoimmune encephalomyelitis (23–26). A recent study revealed a critical role of endothelial cellular A_2A_R-GSK3β signaling in a diet-induced insulin resistance mouse model (14). The above study found that the A_2A_R activation might induce BBB breakdown and cognitive impairment. In contrast, little is known about the effects of A_2A_R on Tau accumulation and BBB integrity in a chronic METH mouse model. We found that A_2A_R was activated in mouse endothelial cells after METH intoxication by the co-staining of CD31 (a marker of endothelial cells) with A_2A_R. Interestingly, the GSK3β was also activated, which was characterized by the phosphorylation of the Y216 site. Next, we inhibited A_2A_R and found that the p-GSK3β level was decreased, indicating that the A_2A_R might trigger GSK3β activation. As the GSK3β is a type of Tau kinase, we tested the endothelial cell p-Tau level by co-staining CD31 and p-Tau. We found that the p-Tau accumulated in the endothelial cells. Based on these results, we concluded that Tau phosphorylation was mediated by A_2A_R-GSK3β signaling in the METH mouse model.

As p-Tau is a type of toxic protein in multiple neurodegenerative diseases (27, 28). The aggregated p-Tau in neurons might induce neuronal morphological changes and functional impairments by affecting microtubule dynamics (29). We speculated that the endothelial cellular p-Tau accumulation might also lead to morphological abnormalities and dysfunction of endothelial cells. As expected, p-Tau accumulation in endothelial cells leads to TJ loss and decreased TJ protein levels in the BBB in the TEM and immunoblotting results, indicating that a compromised BBB integrity occurred in the brain. Recent studies have shown that the BBB breakdown might lead to behavioral abnormalities (30). Interestingly, we found that mice exhibited anxiety- and depression-like behaviors by the FST and the OFT in the METH model. Given the known effects of METH on motor activity, the OFT and FST tests could be influenced by motor function. Thus, we used a dose-escalating schedule of METH exposure, which could minimize the effects of motor dysfunction. Other tests that could not be influenced by motor function should be used to assess the anxiety- and depression-like behavioral test in further studies. Next, we hypothesized that lowering endothelial cellular p-Tau level might alleviate the BBB disruption and behavioral abnormalities induced by METH. We used A_2A_R and GSK3β inhibitors (SCH58261 and lithium chloride, respectively) to inhibit Tau phosphorylation and found that inhibiting A_2A_R-GSK3β signaling diminished the BBB breakdown, reduced TJ protein loss, and mitigated anxiety- and depression-like behaviors induced by METH.

Studies have shown that Tau knockout has no effects on behavioral changes and neuronal dysfunction (17). However, a study showed that knocking out Tau might induce PD pathology through impairment of iron homeostasis (31). Our previous study showed that the motor abilities were not impaired in Tau knockout mice (17). We found that knockout of Tau decreased α-synuclein, which is characteristic of PD in the METH mouse model. In this study, we tested the phenotype of Tau knockout mice after METH exposure. We found that Tau’s lack of expression also alleviated METH-induced BBB disruption, reduced TJ protein loss, and alleviated anxiety- and depression-like behaviors. Notably, the behavioral performances of Tau knockout mice were not comparable to those of wild-type mice. We propose that endothelial Tau hyperphosphorylation triggered endothelial cell pathology, leading to BBB breakdown.

It is notable to increase the limitations of this study. First, we did not use female mice in our study because the female estrous cycle might influence the behavioral tests in the chronic METH model. Second, we did not use specific inhibitors to target endothelial A_2A_R and GSK3β. Third, we did not target endothelial-specific Tau hyperphosphorylation to verify the connection between endothelial Tau and BBB breakdown directly. Endothelial-specific Tau modification should be conducted to test this hypothesis in further studies.

**Figure 6:**
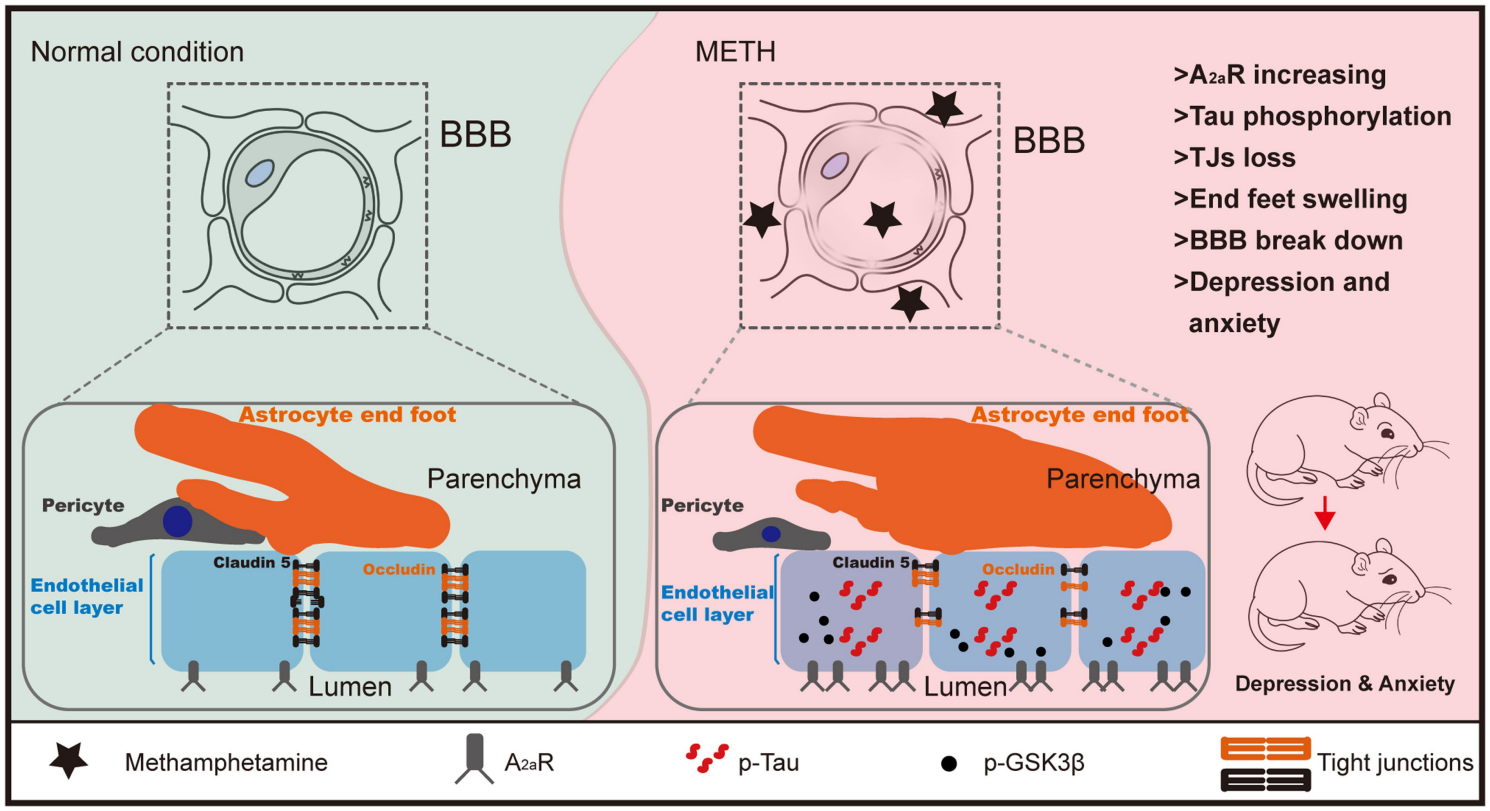
Schematic illustration of mechanisms of METH-induced BBB breakdown and behavioral changes. METH activated the endothelial cellular A_2A_R-GSK3β-Tau signaling pathway, leading to endothelial cellular TJ loss and BBB integrity impairment. The disrupted BBB caused behavioral abnormalities, including anxiety- and depression-like behaviors.

Our findings provided compelling evidence that endothelial cellular A_2A_R-GSK3β signaling was involved in the METH-induced BBB breakdown and anxiety- and depression-like behaviors. Specifically, METH activated the A_2A_R-GSK3β signaling pathway, leading to Tau phosphorylation in endothelial cells. The aggregated p-Tau induced endothelial cell dysfunction and pathology, leading to TJ loss and BBB breakdown. The disrupted BBB caused behavioral abnormalities, including anxiety- and depression-like behaviors. Pharmacological or genetic manipulation of A_2A_R-GSK3β-Tau signaling alleviated BBB disruption and behavioral changes in the chronic METH model (Figure 6). Targeting A_2A_R-GSK3β signaling might have potential effects for application against METH-induced BBB destruction and behavioral abnormalities.

## Data Availability

The original contributions presented in the study are included in the article/supplementary material, further inquiries can be directed to the corresponding author.
